# The Clinical and Cost-Effectiveness of an Individualized Nutritional CAre (INCA) Bundle versus Standard Care for Adults with Pressure Injuries Receiving Home Nursing Services: A Protocol for a Cluster Randomized and Pragmatic Clinical Trial with an Economic Evaluation

**DOI:** 10.3390/nu16020299

**Published:** 2024-01-18

**Authors:** Alvin Wong, Precilla Lai, Hui Hsien Chong, Christopher Tsung Chien Lien, Nicholas Graves

**Affiliations:** 1 Department of Dietetics, Changi General Hospital, 2 Simei Street 3, Singapore 529889, Singapore; 2 Department of Nursing, Home Nursing Foundation, 490 Lorong 6 Toa Payoh, #05-10, Singapore 310490, Singapore; precilla.lai@hnf.org.sg; 3 Department of Geriatric Medicine, Changi General Hospital, 2 Simei Street 3, Singapore 529889, Singapore; 4 Health Services and Systems Research, Duke-NUS Medical School Singapore, 8 College Road, Singapore 169857, Singapore

**Keywords:** pressure injury, nutrition support, dietetic, home nursing

## Abstract

Background: Pressure injuries (PIs) represent a significant healthcare challenge in Singapore among the aging population. These injuries contribute to increased morbidity, mortality, and healthcare expenditure. Existing research predominantly explores single-component interventions in hospital environments, often yielding limited success. The INCA Trial aims to address this research gap by conducting a comprehensive, cluster randomized controlled trial that integrates education, individualized nutritional support, and community nursing care. This study is designed to evaluate clinical and cost-effectiveness outcomes, focusing on PI wound area reduction and incremental costs associated with the intervention. Methods: The INCA Trial employs a two-group, non-blinded, cluster randomized, and pragmatic clinical trial design, recruiting 380 adult individuals (age ≥ 21 years) living in the community with stage II, III, IV, and unstageable PI(s) who are receiving home nursing service in Singapore. Cluster randomization is stratified by postal codes to minimize treatment contamination. The intervention arm will receive an individualized nutrition and nursing care bundle (dietary education with nutritional supplementation), while the control arm will receive standard care. The 90-day intervention will be followed by outcome assessments extending over one year. Primary outcomes include changes in PI wound area and the proportion of participants achieving a ≥40% area reduction. Secondary outcomes include health-related quality of life (HRQOL), nutritional status, and hospitalization rates. Data analysis will be conducted on an intention-to-treat (ITT) basis, supplemented by interim analyses for efficacy and futility and pre-specified sensitivity and subgroup analyses. The primary outcome for the cost-effectiveness analysis will be based on the change to total costs compared to the change to health benefits, as measured by quality-adjusted life years (QALYs). Discussion: The INCA Trial serves as a pioneering effort in its approach to PI management in community settings. This study uniquely emphasizes both clinical and economic outcomes and melds education, intensive dietetic support, and community nursing care for a holistic approach to enhancing PI management.

## 1. Background

A pressure injury (PI) is defined as a “localized injury to the skin and/or underlying tissue usually over a bony prominence, as a result of pressure, or pressure in combination with shear” [1]. In Singapore, the majority of adults with stage III to IV PIs were community-acquired, [2] with a prevalence of 6% to 29% reported [3]. An incidence rate of 505 (95% CI: 493–517) per 100,000 persons has been reported, and it has been observed to increase with age, with the sharpest rise observed in individuals aged 80 years and above, at 3647 (95% CI: 3530–3766) per 100,000 persons [3,4].

PI(s) are associated with increased morbidity and mortality. Zhan and Miller [5] reported 3.98 days of extra hospitalization (*p* < 0.001) and 7.23% excess mortality (*p* < 0.001). Locally, one-fifth (20.0%) of patients had two or more PI-related readmissions for the index wound [6]. On subsequent follow-up one year later, the all-cause mortality rate was 14.3%, with 26.8% of patients institutionalized upon discharge from the tertiary hospital [6]. 

The most recent 2014 Cochrane review found no clear evidence that nutritional interventions reduce/heal PI(s) [7], but newer studies in recent years indicated potential benefits of using specialized nutritional feeds containing specific amino acids, such as arginine and leucine, as well as antioxidants, such as Vitamins C and E [8,9,10,11]. 

Integrated care is still in its development stages in Singapore and Asian countries, in comparison to the countries in North America and Europe [12]. Patients with PIs in Singapore are routinely reviewed by dietitians in acute care settings but rarely in the community upon discharge. This may be related to the failure to return for outpatient clinic reviews due to immobility and social reasons [13]. Furthermore, there is limited access to community dietitians and funding for dietetic review or nutritional support [14]. Most patients and their caregivers/family members also do not receive adequate nutritional education on PI treatment and prevention, as it is not a standard procedure in most healthcare settings. As a result, the majority of patients or caregivers do not have the necessary nutritional knowledge for PI management. 

Early nutritional studies on PI wound healing generally implement simple [15] single-component interventions using specialized medical nutritional supplements [16,17,18,19] or primarily concentrate on hospitalized patient populations, where the intervention period is inadequate to deliver a positive outcome [19,20]. Recently, a cluster randomized controlled trial (RCT) of 1600 hospitalized patients using a complex multi-component PI prevention intervention (posters/DVD and face-to-face education) showed a statistically non-significant reduced hazard ratio for new PI(s) developed [21,22]. However, one of the limitations of this study was the lack of individualized nutritional intervention with supervision.

To address this limitation and build on the body of work performed, we intend to conduct a cluster randomized controlled trial, implementing a multi-component PI care bundle intervention consisting of education, dietetic support with medical nutritional supplementation, and trained home care/community nurses in nutrition care, for patients in the community with existing PI(s) (stages II, III, IV, and unstageable). 

The objectives of this study are to evaluate the clinical and cost-effectiveness of the multi-component PI care bundle intervention on pressure injury-related outcomes. The primary outcomes for the cluster randomized controlled trial were the change in PI area from the baseline in the intervention and study period and the proportion of participants with a 40% or greater reduction in the wound area. The primary outcomes for the economic evaluation of the trial are the change to total costs to account for incremental costs of the trial intervention for the change to health benefits, measured by quality-adjusted life years (QALYs), and the primary endpoints of the clinical trial.

## 2. Methods/Design

Standard protocol items for reporting cluster RCT were followed using the Spirit statement [23] and the SPIRIT-OUTCOMES extension [24], with the Template for Intervention Description and Replication (TIDieR) [25] checklist used to guide the description of the study intervention. Refer to the Supplementary Material for the completed checklists.

### 2.1. Study Design

We intend to perform a two-group, non-blinded, cluster randomized, and pragmatic clinical trial with the recruitment of 380 subjects (190 per arm). The duration of the intervention will be 90 days, with data collection of outcomes performed over a one (1)-year period. 

### 2.2. Study Population and Setting

Adults (age ≥ 21 years of any gender) living in the community with stage II, III, IV, and unstageable PI(s) who are receiving home nursing services in Singapore (28 districts comprising 82 postal codes) were eligible. 

### 2.3. Inclusion and Exclusion Criteria 

Patients will be considered eligible if they are able to provide written informed consent (patient or legal guardian). The patients can be on oral and/or enteral nutritional support. The exclusion criteria for the trial are presented in Table 1.

### 2.4. Sample Size Estimation

The sample size calculation is based on the results reported by Cereda et al. [17], where an odds ratio (OR) of 2.0 for ≥ 40% improvement in wound area on patients receiving a specialized nutritional supplementation containing arginine is used. To have 80% power to detect this difference with a 2-tailed type I error of less than 5%, we require at least 144 subjects per arm. After assuming a 20% mortality and 10% withdrawal rate (based on a Cereda et al. [17] study of 16% mortality and 6% withdrawal and a Chaboyer et al. [22] study of 8% loss to follow-up), we plan to enroll a total of 380 subjects (190 per arm). As we will be undertaking an intention-to-treat (ITT) analysis, anticipated adherence will be one of the outcome measurements to determine if the intervention (education and supplementation) is acceptable to the participants (Supplementary Material Figure S1).

### 2.5. Randomization

We will randomize patients into two groups using a cluster randomization method, where the patient population is stratified into two groups based on the locality (postal code) and a random 1:1 block allocation of postal codes (under the care of nursing teams) to either intervention or control group. The allocation of the groups will be concealed until participation is fully confirmed. 

The primary reason for cluster randomization is to limit treatment contamination between the intervention and control groups. As the nurses from home nursing services are allocated to the care of multiple patients within the same neighborhood (based on postal code), individual randomization may increase the risk of the control group learning about the nutritional intervention (type II error) from interactions with the assigned home care nurse.

We will engage a statistician who is not associated with recruitment to generate a random number list to determine the order in which the recruiting staff will visit the specified postal codes for participant recruitment. Postal codes with low residential density in Singapore (limited cluster size) may be excluded or combined with other postal codes if feasible. 

We aim to recruit 8 to 10 subjects per week and finish recruitment in 35 to 50 weeks. We determined this rate of recruitment as there are currently 500 to 600 individuals under home nursing care with known PI(s), with the expectation that 20% to 30% of individuals will not meet the selection criteria. For patients with multiple wounds, all the PI(s) in stages II and above will be included in the analysis.

### 2.6. Recruitment 

For each postal code, the trial statistician will develop a randomization schedule to determine the order of recruiters approaching participants for recruitment. This will ensure that all the postal codes are fairly represented in the recruitment phase. The recruiters will screen the eligibility of potential participants and approach them with verbal and written information to explain the study before seeking consent. Consent/assent procedures (e.g., by whom, how, and under what conditions will a subject have consented) are presented in the Supplementary Material. Due to the profile of the subjects and caregivers and the frequency of clinical visits, a 21-day window period will be implemented from consent to the baseline visit.

### 2.7. Blinding and Allocation Concealment

Subjects will not be blinded to group allocations due to the nature of this study, which is not a placebo-controlled trial but rather a real-world pragmatic study. Subjects, research dietitians, and nursing staff will be aware that they are in a study to examine the effects of nutritional intervention on pressure injury healing rates. The nursing personnel responsible for measuring wounds and gathering data will not be blinded, given their role in conducting follow-up with the participants as a component of clinical care. However, they will be randomized to ensure they do not attend to both the intervention and control groups. The research dietitians who are not part of the main study team will only have interaction with the intervention group participants, and they are blinded to the measurement of primary outcomes. The trial statistician will be blinded to the group allocation.

### 2.8. Training for Home Care Nurses and Research Dietitians and Treatment Fidelity

Before study recruitment, home care nurses will undergo training sessions by the Principal Investigator (Dietitian) on the topics of (A) *Nutritional Screening and Assessment* (3 h), (B) *Management of Malnutrition* (2 h), (C) *Nutritional Interventions for Pressure Injury* (3 h), and (D) *Study Protocol Familiarization* (2 h). This will be carried out in eight sessions over three months. The training will include formal teaching with education materials and self-learning modules/reading materials, with informal discussions post-training to engage with the nursing staff. This will ensure nursing staff understand the importance of individualized nutritional support for wound healing in addition to the usual nursing procedures. 

The research dietitians, research coordinators, and nurses will undergo group-specific training tailored to their role in the research to ensure consistent protocol implementation and data collection across all clusters. The research coordinators and nurses will be provided with standardized data collection forms and plans for dealing with fidelity issues about the interventions. Participants in both the intervention and control groups will receive identical information and instructions regarding the study, except for the actual intervention. The intervention group will be told they will receive extra nutritional support intervention and supplementation, while the control group will be told to receive the usual nutritional care based on the most recent recommendation from their usual healthcare provider.

### 2.9. Intervention

Based on the latest guidelines, the intervention group will receive an educational pamphlet on nutritional and wound care for participants/families, with an in-depth explanation by trained nursing staff and a research dietitian. The trained nurses will reinforce nutritional education (adequate energy protein/fluid intake and compliance to consumption of supplements) on visits without a dietitian. The nutritional intervention provided will be based on the “The European Pressure Ulcer Advisory Panel, the National Pressure Injury Advisory Panel, and the Pan Pacific Pressure Injury Alliance Prevention and Treatment of Pressure Ulcers/Injuries: 2019 Clinical Practice Guideline” [1,33].

A dietetic consultation at the baseline, day 30, and day 60 of the intervention will be performed by an experienced and trained research dietitian on optimizing nutritional intake to meet caloric/protein requirements, along with additional high protein high energy (HPHE) supplementation (commercial ONS) prescribed for participants who do not meet nutritional requirements (25–35 kcal/kg/d and 1.2–2.0 g protein/kg/d) [1,33]. The dietetic consultation will be performed via face-to-face home visits and/or teleconsultation (depending on participant and caregiver preference). Adjustments to the enteral feeding regimen will be made for patients who did not see a dietitian during their last hospital admission. 

A specialized nutritional supplement for wound healing (Arginaid, Nestle, Vevey, Switzerland) containing 4.5 g arginine, 156 mg Vitamin C, 40.9 mg alpha-tocopherol equivalents (Vitamin E), and 30 kcal will be administered twice a day via oral ingestion or tube feeding (mixed in 100 mL water) for 12 weeks (14 servings per week). Adherence to supplementation will be determined by recording the number of leftover products by participants or their caregivers, with confirmation of intake during the research dietitian or nurse visit on days 30, 60, and 90. 

### 2.10. Control 

The control group will receive an educational pamphlet on nutritional and wound care for patients/families based on the latest nursing and nutritional guidelines [1,33], with an in-depth explanation by trained nurses. The trained nurses will provide reinforcement of nutritional education during the planned visits as per usual practice over the 90 days. Standard care and follow-up, as per hospital care plans, with the home care nurse contacting the hospital dietitian-in-charge of the subject for verbal advice as per usual practice, will be followed. HPHE supplementation and dietary advice to meet nutritional requirements will be based on previous recommendations from the hospital dietitian-in-charge or clinician.

## 3. Outcome Measures

We included objective measures (area, wound healing rate, and proportion of participants with wound reductions) for the primary outcome measurements, as these measurable outcomes will be used for the economic evaluation component of this study. 

### 3.1. Primary Outcome Measurements

1. Change in Pressure Injury Area from the Baseline: Measurement of the alteration in the surface area of the identified pressure injury (PI) at identified time points;
2. Proportion of Participants with 40% or Greater Area Reduction: Calculation of the percentage of participants who exhibit a reduction in the PI area by at least 40% at the same time points.

For participants with multiple PIs, the most severe or largest same-stage PI will be selected for primary analyses. Data from additional PIs will contribute to secondary analyses. Assessments and treatment documentation will be conducted during scheduled home nursing visits. Both objective and patient-reported outcome measures are used for the secondary outcome measurements to ensure that crucial additional information is collected from the participant’s perspective. 

### 3.2. Secondary Outcome Measurements

1. Proportion with Increased Severity in Pressure Injury Stages: The percentage of participants whose pressure injury progresses in severity (PI stage and area of wound);
2. Proportion with Complete Pressure Injury Healing: The percentage of participants whose PIs fully heal;
3. Proportion with Improvement in Health-Related Quality of Life (HRQOL): The percentage of participants showing enhancements in HRQOL, as measured by the standardized instrument EQ-5D-5L;
4. Proportion with Nutritional Status Improvement: The percentage of participants with measured nutritional advancements;
5. Change in HRQOL (EQ-5D-5L Utility Values and VAS): The measured alteration in HRQOL utilizing EQ-5D-5L utility values and Visual Analog Scale (VAS);
6. Incidence of New PIs: The number of new PIs during the study period;
7. Incidence of PI Wound Infections: The number of new infections in PI wounds during the study period;
8. Mortality Rate: The death rate among participants during the study time frame;
9. Unplanned Hospital Admissions and Length of Stay (LOS): The frequency and duration of unexpected hospital admissions within the participant group.

All the study data and outcome measurements listed in Table 2 will be recorded using the Research Electronic Data Capture (REDCap) system [34]. Sociodemographic data will be collected based on recommendations by a local systematic review of healthcare utilization in urban Singapore [35]. 

A window period of ±14 days will be implemented for all assessments and outcome measurements. If an outcome measurement is interrupted (e.g., malfunction of wound imaging equipment, secure cloud server downtime, or assessors unable to visit on the day of assessment due to participant’s request for postponement), the assessor will re-attempt the measurement within the window period. Any outcomes measured outside of this window period will be considered a protocol deviation. 

## 4. Discontinuation of Study for Subjects by the Research Team

The study team can discontinue the subject from the study, and possible reasons for discontinuation from the study include:

(1) Intolerance to oral nutritional supplements and/or specialized wound supplements such as nausea, vomiting, bloatedness, and diarrhea;
(2) Allergic reaction to specialized wound supplements;
(3) Frequent readmission to hospitals for wound infections that cannot be managed in the home care setting;
(4) Non-compliance to supplementations or dietary advice provided;
(5) Non-compliance to wound management advice;
(6) Worsening of the wound requiring surgical intervention;
(7) New diagnosis of diseases included in the exclusion criteria;
(8) New diagnosis of infectious diseases (such as COVID-19) that requires the subject to be isolated for >7 days and prevents the research team from having access to the subject.

## 5. Statistical and Analytical Plans

We will implement an intention-to-treat (ITT) analysis, meaning all subjects randomized to either the intervention or control groups will be analyzed. Subjects who withdraw or are lost to follow-up will have their data analyzed up to their last recorded home nursing visit.

### 5.1. Outcome Measures

1. Dichotomous Outcomes: The frequency of outcomes will be determined, and we will utilize time-to-event analyses. The hazard ratios (HRs) along with their 95% confidence intervals (CIs) will be determined using a stratified Cox proportional hazards model.
2. Continuous Outcomes: The differences between the intervention and control groups, along with their 95% CIs and corresponding *p*-values, will be documented.

Both patient-level and cluster-level data will be analyzed. We will address any imbalances in individual data by adjusting statistically using a mixed-effects Cox regression model performed by a statistician blinded to the group allocation. 

### 5.2. Sensitivity Analyses

To ensure the robustness of our findings, sensitivity analyses will be conducted. Estimates will be adjusted considering pre-specified factors derived from the literature or known PI risk factors, such as age, BMI, nutritional status, wound severity, comorbidities, and socioeconomic status.

### 5.3. Subgroup Analyses

A planned subgroup analysis will be performed by targeting specific nutritional status and intake variables, route of intake (oral versus enteral), staging of PIs, gender, age group, compliance to intervention, and functional status. 

### 5.4. Secondary Analyses

Per-protocol analyses planned include the proportion of wounds that will be executed, excluding subjects who:

1. Suffer from wound infections;
2. Show non-compliance to the treatment;
3. Dropout from the trial;
4. Die during the trial period.

### 5.5. Interim Analyses

We intend to conduct interim analyses for efficacy and futility when data are finalized for subsets of 100 and 200 subjects. Based on these interim findings, we might reassess our sample size to maintain the power required to detect a significant difference in the primary outcome between our two study groups. Sample size re-estimation may be performed to evaluate the power to detect a statistically significance difference in primary outcome between the intervention group and the control group based on an interim analysis. 

## 6. Economic Evaluation Protocol and Analytic Plans

As part of the research, a planned micro-costing study and cost-effectiveness analysis (CEA) alongside the cluster randomized controlled trial will be undertaken from the payer and healthcare system perspective. The economic evaluation protocol is developed in accordance with the CHEERS 2022 (Consolidated Health Economic Evaluation Reporting Standards 2022) [36] and international cost-effectiveness analysis guidelines [37,38,39].

### 6.1. Micro-Costing 

Micro-costing will be performed on a subsample of participants (n = 40; 10% of the sample population) over a 4-week period to supplement cost data for the CEA. This sample size will be 10% of the trial cohort and is deemed sufficient to represent the mean and distribution of resource use in the study population. Detailed data on resource use related to intervention will be collected by the home care nurses and research dietitians. We will only include direct costs for the micro-costing; that is, the resources directly consumed in the treatment (Supplementary Material). 

### 6.2. Cost-Effectiveness Analysis 

The primary outcome will be based on an intention-to-treat (ITT) principle for the CEA. The change to total costs will be compared to the change to health benefits, as measured by quality-adjusted life years (QALYs). Total costs will be estimated by considering the extra costs of the intervention and the change to the use of health services in subsequent time periods. QALY outcomes will be estimated using data from the EQ5D-5L, and the change due to the intervention will be estimated. Uncertainty will be included in probabilistic sensitivity analysis.

The incremental mean costs will be estimated from participants in both groups based on resource unit cost multiplied by resource utilization. The change to total costs and QALYs will be considered on the cost-effectiveness plane and analyzed quantitatively to estimate the probability that the adoption of intervention is a good decision against maximum willingness to pay thresholds for marginal health benefits, such as QALYs. Further details of the economic evaluation protocol are available in the Supplementary Material.

## 7. Trial Status

Recruitment started in October 2023.

## 8. Data Collection and Management

### 8.1. Data Collection 

Data will be collected by the nurses (for both groups) and the research dietitian (for the intervention group only) during each planned visit (Table 2). This will be carried out at the baseline, 30 days, 60 days, 90 days, and 12 months for the wound and nutritional data, hospital admissions, mortality, and health-related quality of life (HRQOL). The study schedule of enrolment, interventions, and assessments follows the recommendations from the SPIRIT statement [23] and the SPIRIT-OUTCOMES extension [24], as shown in Table 3.

### 8.2. Data Management

Data collected during the research will be kept strictly confidential and accessed only by delegated research team members. Data collected will be delinked, coded, and de-identified. Only de-identified data will be sent to the statistician for statistical analysis and to the health economist for economic evaluation. The data will be kept for three [3] years post-completion of the trial and will be destroyed and deleted subsequently. Methods may include overwriting data with a series of characters or reformatting the disk (destroying everything on it). If unable to overwrite data, we will consider pulverizing the hard disk to destroy hard disk data based on existing institutional research board guidelines.

### 8.3. Management and Safety

The investigator(s)/institution(s) will permit study-related monitoring, audits, and/or Centralised Institutional Review Board (CIRB) review and regulatory inspection(s), providing direct access to source data/documents to ensure compliance with the relevant data protection legislation. A combination of paper and electronic data will be collected for this study. All data recorded on paper will be handled, transferred, and stored securely. Paper data will be stored in the investigator site file for the duration of the study in a locked cupboard in a locked room. Data from paper records will be uploaded digitally by a delegated member of the local research team. No personal identifiers will be collected in study questionnaires.

Only related severe adverse events (SAEs) will be reported to the CIRB. Related means there is a reasonable possibility that the event may have been caused by participation in the research. The investigator is responsible for informing the CIRB after first knowledge that the case qualifies for reporting. Follow-up information will be actively sought and submitted as it becomes available. Related adverse events (AEs) will not be reported to the CIRB. However, the investigator is responsible for keeping a record of AE cases in the study site file.

We will provide an identification and description of individuals responsible for monitoring the trial, their roles, qualifications, and the frequency of the monitoring activities. We will also include a description of any specific events that would preclude a participant from continuing the intervention. The potential risks and the measures in place to protect participants against foreseeable risks are presented in the Participant Information Sheet and Consent form (Supplementary Material). 

Mechanisms are in place to protect subject privacy (e.g., interviews will take place in a private room, and the results of testing data will be shared with the participant’s legally authorized representative using secure means of communication between investigators and participants). A description of the data security in place to protect the confidentiality of the data is available. Study-stopping rules for the study are in place. The study will be stopped immediately if a serious adverse event that occurred is suspected to be related to the study.

## 9. Discussion

Pressure injuries are a complex and prevalent healthcare concern, particularly in community settings. Existing nutritional studies on PI wound healing have primarily focused on single-component interventions or hospital-based populations, often lacking individualized dietary support [17,19,20,22]. Recent trials have explored multi-component interventions but have shown statistically non-significant results [20,22]. This study addresses these gaps by implementing a comprehensive PI care bundle intervention, including education, dietetic support with medical nutritional supplements, and trained home care/community nurses in nutrition care. The primary objectives are to evaluate the clinical and cost-effectiveness of the intervention on PI-related outcomes. Considering the unique healthcare landscape, the focus on community settings in Singapore adds to the study’s significance.

To our knowledge, the INCA Trial is the first and largest trial attempting a complex intervention on PIs in a community setting. The INCA Trial falls under the category of effectiveness research [40]. Effectiveness research seeks to provide a comprehensive representation of the target study population cohort while contrasting novel interventions with standard clinical practices. Consequently, the findings will be instrumental in informing clinical judgments, thereby enhancing healthcare delivery’s quality, efficacy, and cost-effectiveness.

## 10. Limitations 

The blinding of participants is not feasible for this trial due to the nature of the intervention. While the non-blinded design may introduce bias in outcome assessment [41], care has been taken to mitigate bias through cluster randomization and ensuring that outcome assessors are assigned to only one study group over the intervention period. 

The 90-day intervention period may not capture long-term effects on wound healing, potentially missing essential insights into the sustainability and long-term impact of the intervention. Hence, outcome measurements at 6 months and 1 year and an economic evaluation alongside the cluster RCT have been planned to determine if this intervention period is adequate.

The focus on community settings in Singapore may also affect the generalizability of the results to other healthcare systems. However, as pressure injury management requires an extended period of care and is generally not managed within a fixed healthcare setting and environment, the findings will contribute to evidence-based management, improving patient outcomes and healthcare cost-effectiveness. The study’s potential impact on policy and practice, considering the unique healthcare financing and reimbursement landscape in Singapore for nutritional intervention [14], adds to its relevance and importance.

## 11. Conclusions

This trial is designed to provide insights into the effectiveness of a comprehensive PI nutrition and nursing care bundle intervention. The focus on both clinical and economic aspects sets this study apart from previous interventions. The integration of education, intensive dietetic support, and nutrition-trained home care/community nurses in nutrition care offers a holistic approach to PI management.

## Figures and Tables

**Table 1:** Exclusion criteria for the INCA Trial.

	Exclusion Criteria
1.	Septicaemia.
2.	Poorly controlled diabetes (*glycated hemoglobin level > 8.5%)* [20,26].
3.	Consumption of supplements will lead to fluid intake in excess of fluid restriction for the following conditions: Advanced renal disease not on dialysis (KDIGO [21,22] Stage G4 with an eGFR of 15–29 mL/min/1.73 m^2^ and Stage G5 with an eGFR of less than 15 mL/min/1.73 m^2^); Heart failure with reduced ejection fraction [27]; Advanced decompensated alcoholic and non-alcoholic liver cirrhosis [28,29].
4.	Current neoplastic disease or last chemotherapy or radiotherapy less than one year ago.
5.	Currently on immunosuppressive therapy.
6.	Palliative with *a lifespan of ≤ 3 months.*
7.	Known allergy reaction to L-arginine or phenylketonuria.
8.	Presence of an infected wound *(if it is the only pressure injury present on the participant).*
9.	Untreated diagnosed osteomyelitis [18,19,30,31,32].
10.	Pregnant women and children (there is limited evidence on the use of arginine in these groups).

**Table 2:** Outcomes and the methods of measurement to be used in the cluster RCT.

Type	Outcome	Outcome Description	Measurement Method	Data Collection Time Point (t) and Time Frame	Performed by
Primary	Wound	Change in the area of pressure injury (cm^2^) from the baseline	Assessed by the change in wound area ^#^ from the baseline to the follow-up time points and measured with a 3D wound imaging device at the time of follow-up.	t_0_ baseline, t_1_ 30 days, t_1_ 60 days, t_3_ 90 days, t_4_ 6 months, and t_x_ 1 year Time frame: 1 year	Nurse
		The proportion of participants with ≥40% area reduction	Assessed by the number of participants with wound area ^#^ reduction ≥ 40% at the time of follow-up versus the baseline.	t_1_ 30 days, t_1_ 60 days, t_3_ 90 days, t_4_ 6 months, and t_x_ 1 year Time frame: 1 year	Nurse, CRC
Secondary	Wound	The proportion of participants with complete wound healing of the main wound	Assessed by the number of participants with complete healing of a wound ^#^, determined by clinical assessment and pressure injury staging.	t_1_ 30 days, t_1_ 60 days, t_3_ 90, t_4_ 6 months days, and t_x_ 1 year Time frame: 1 year	Nurse, CRC
		The proportion of participants with increased severity of pressure injury (PI stage)	Assessed by the number of participants with increased severity of wound ^#^ at follow-up. The severity of pressure injury is determined by the increase in pressure injury staging or the increase in the area of the wound if pressure injury staging remains the same at the follow-up time points.	t_1_ 30 days, t_1_ 60 days, t_3_ 90, t_4_ 6 months, and t_x_ 1 year Time frame: 1 year	Nurse, CRC
		The proportion of participants with a new wound infection	Assessed of the number of participants with new wound infection(s) at follow-up who are clinically diagnosed with confirmation from blood tests (e.g., C-Reactive Protein CRP, renal, and liver function tests).	t_1_ 30 days, t_1_ 60 days, t_3_ 90 days, t_4_ 6 months, and t_x_ 1 year Time frame: 90 days	Nurse
	Nutritional	Change in nutritional status	Assessed using the Global Leadership Initiative on Malnutrition (GLIM) criteria to determine the severity of malnutrition. Change in the nutritional status determined by the direction of the nutritional status shift from the baseline to follow-up.	t_1_ 30 days, t_1_ 60 days, t_3_ 90 days, and t_x_ 1 year Time frame: 1 year	Nurse, Dietitian
		Change in nutritional intake	Assessed by the change in average of energy, protein, and selected micronutrient intake derived from 3DFR. Three-day food records will be filled by the participants or caregivers at selected time points. If the family or participant is unable to fill out the record, photographs of meals can be taken by the subject or caregiver and sent to the CRC. Trained personnel will use food composition analysis software (DietPlan 7, Forestfield Software Ltd., UK) to determine the intake, adjusted to per unit kilogram weight of the participant The energy, protein, and micronutrient intake of the study subjects on enteral tube feeding will be determined by a calculation of the goal feeding regimen that the study subject has been prescribed prior to the start of the study.	t_0_ baseline, t_1_ 30 days, and t_1_ 60 days Time frame: 60 days	Dietitian, Nurse
	Quality of Life	Change in HRQOL	Assessed by the change in EQ5D-5L VAS and utility scores at follow-up from the baseline for the overall population and age categories, if appropriate, where changes in scores are expressed as mean differences or standardized mean differences.	t_0_ baseline, t_3_ 90 days, t_4_ 6 months, and t_x_ 1 year Time frame: 1 year	Dietitian, Nurse, CRC
	Clinical	Mortality	Assessed by the occurrence and time-to-event of all-cause mortality during the study period.	t_1_ 30 days, t_1_ 60 days, t_3_ 90 days, and t_x_ 1 year Time frame: 1 year	Nurse, CRC
		Unplanned hospital admissions	Assessed by the occurrence and time-to-event of one or more unplanned hospital admissions during the study period.	t_1_ 30 days, t_1_ 60 days, t_3_ 90 days, and t_x_ 1 year Time frame: 1 year	Nurse, CRC
Others	Pre-specified Outcome	Adherence to nutrition Supplementation intake	Assessed by the number of participants with >75% consumption of oral nutritional supplements and Arginaid, where the servings of products consumed are counted with confirmation of intake during follow-up.	t_1_ 30 days, t_1_ 60 days, and t_3_ 90 days Time frame: 1 year	Dietitian, Nurse, CRC
		Wound depth	Measured (millimeters) using sterile forceps and rulers at follow-up.	t_0_ baseline, t_1_ 30 days, t_1_ 60 days, 90 t_3_ days, and t_x_ 1 year Time frame: 1 year	Nurse
		Hospital length of stay	Assessed by the number of days for each unplanned hospital admission.	If available: t_x_ 1 year Time frame: 1 year	Nurse, CRC
		Wound duration and time-to-heal	Measured by days to complete healing for participants with complete wound healing. Additional interaction of time with intervention and other covariates will be included and tested	t_x_ 1 year Time frame: 1 year	Investigators
		Frequency of unplanned hospital admissions	Assessed by the frequency of occurrence per participant and the sum of unplanned hospital admissions of all participants.	t_x_ 1 year	Investigators

^#^ The data from the measurement of the pressure injury with the highest severity will be used in the analysis for the primary endpoint. If the wound of the highest severity is deemed unmeasurable (e.g., too small of a surface area), we will select the wound with the next highest severity. CRC: clinical research coordinator; HRQOL: health-related quality of life; PI: pressure injury.

**Table 3:** Study schedule of enrolment, interventions, and assessments.

	STUDY PERIOD							
	Enrollment	Allocation	Post-Allocation					Close-Out
TIMEPOINT	*−7 to −1d* −*t_0_*	Baseline *t_0_*	*30d t_1_*	*60d t_2_*	*90d t_3_*	*6m t_4_*		*1y* *t_x_*
**ENROLMENT:**								
***Eligibility screen***	X							
***Informed consent***	X							
***Cluster randomization***	X							
***Allocation***		X						
**INTERVENTIONS:**								
***Intervention***			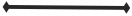					
***Control***			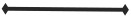					
**ASSESSMENTS:**								
***Sociodemographic***	X	X						
***Medical history***	X	X						
***QoL (EQ5D-5L)***		X			X	X		X
***Wound (new, infection, 3D imaging, depth, and stage)***		X	X	X	X	X		X
***Nutritional status (GLIM)***		X	X	X	X			X
***Nutritional*** ***intake (3DFR)***		X		X				
***Unplanned hospitalization and LOS***			X	X	X	X		X
***Complications***			X	X	X	X		X
***Mortality***			X	X	X	X		X
***Nutritional*** ***intake (compliance)***			X	X	X			

## Data Availability

Not applicable.

## Supplementary Materials

The following supporting information can be downloaded at https://www.mdpi.com/article/10.3390/nu16020299/s1, Figure S1: Informed Consent and Determining Legal Representative in Adults Lacking Capacity; Table S1: Sample size calculation for INCA Trial; Table S2: Economic Evaluation Protocol Summary [42,43].

## Abbreviations

AEs	Adverse Events
CEA	Cost-Effectiveness Analysis
CI	Confidence Interval
CIRB	Centralised Institutional Review Board
CRC	Clinical Research Coordinator
eGFR	Estimated Glomerular Filtration Rate
HPHE	High Protein High Energy
HRQOL	Health-Related Quality of Life
ITT	Intention-To-Treat
LOS	Length of Stay
ONS	Oral Nutritional Supplement
OR	Odds Ratio
PI	Pressure Injury
QALYs	Quality-Adjusted Life Years
RCT	Randomized Controlled Trial
TIDieR	Template for Intervention Description and Replication
SAEs	Severe Adverse Events
