# Supplementary material for: The Clinical and Cost-Effectiveness of an Individualized Nutritional CAre (INCA) Bundle versus Standard Care for Adults with Pressure Injuries Receiving Home Nursing Services: A Protocol for a Cluster Randomized and Pragmatic Clinical Trial with an Economic Evaluation
Source: Nutrients. 2024 Jan 18;16(2):299. doi: 10.3390/nu16020299 (PMC10819700; doi:10.3390/nu16020299)
Supplement: Supplementary file 1 [file nutrients-16-00299-s001.zip › nutrients-2820833-supplementary.pdf]

Supplementary Material

A. Statistical Analysis and Sample Size Calculation

Incidence Rate Calculation

The incidence will be calculated by the division of all-cause mortality during the follow up timeframe by the number of the number of participants initially at risk at baseline.

Sample Size Calculation

Table S1. Sample size calculation for INCA Trial [43,44]

**Sample Size:X-Sectional, Cohort, & Randomized Clinical Trials**			
Two-sided significance level(1-alpha):			95
Power(1-beta, % chance of detecting):			80
Ratio of sample size, Unexposed/Exposed:			1
Percent of Unexposed with Outcome:			55
Percent of Exposed with Outcome:			71
Odds Ratio:			2
Risk/Prevalence Ratio:			1.3
Risk/Prevalence difference:			16
	**Kelsey**	**Fleiss**	**Fleiss with CC**
Sample Size - Exposed	144	143	155
Sample Size-Nonexposed	144	143	155
Total sample size:	288	286	310

References
Kelsey et al., Methods in Observational Epidemiology 2nd Edition, Table 12-15
Fleiss, Statistical Methods for Rates and Proportions, formulas 3.18 &3.19
CC = continuity correction
Results are rounded up to the nearest integer.
Print from the browser menu or select, copy, and paste to other programs.

Results from OpenEpi, Version 3, open source calculator--SSCohort

B. Economic Evaluation Methodology

Healthcare Resources Utilisation

The healthcare resource utilisation will be collected alongside the cluster RCT. Based on an earlier systematic review,[35] we identified a list of relevant healthcare resource items. The resources that are monitored will include additional medical nutritional supplements prescribed, medications (antibiotics for infected wounds),

wound dressing materials (estimated daily use), operative and non-operative procedures for treatment of PI (investigative scans, pressure redistribution mattresses, negative pressure wound therapy, surgical debridement,), scheduled blood tests by home nursing services, health professional services (nursing care, dietitian face-to-face /or telehealth services, medical outpatient and emergency department visits specific for PI-related problems. For missing resource utilisation data, appropriate statistical methods will be used, depending on the type and distribution of the missing data.[36]

Unit Costs

Data collection for the costing study is anticipated to start at recruitment mid-point of the main trial, and accounting data retrieved at the end of the trial. Direct medical costs related to the treatment of pressure injuries will be assessed. We will not be addressing the indirect costs (e.g., time costs or production loss from work among individual and/or family members) as caregiving depends mainly on external resources. Additionally, the level of dependence for pressure injury care is likely constant during the intervention period. Resource utilization during the trial will be recorded daily.

Costs associated with debridement performed before study entry will not be considered. In the event that we are unable to obtain the actual costs, we will use the benchmark fees for surgical debridement available from the Ministry of Health (Singapore).[37] Daily fees for the local healthcare facilities will also be used if participant is admitted for reasons related to pressure injury complications. The hourly rate or per service visit fees for nursing, dietitian and medical visits will be used for the estimation of expenses for health professional services. Prices will be reported in Singapore Dollars (Year 2023). Discounting is not required as the intervention will be carried out in 3 months, and prices of public healthcare utilisation are unlikely to change.

Table S2. Economic Evaluation Protocol Summary

	**Summary of Economic Evaluation Framework**
**Hypothesis/Question**	The use of PINCB when compared with standard care, is cost-effective in the treatment of pressure injuries (wound healing, wound area reduction and preventing new wounds) and other clinical outcomes (wound infection, mortality, and hospitalisation), in home-nursing adults with pressure injuries stage II and above.
**Background Objectives**	The primary outcome will be based on an intention-to-treat (ITT) principle for the CEA. The change to total costs will be compared to the change to health benefits, as measured by quality adjusted life years (QALYs).
**Target Population**	Home-nursing adults with pressure injuries stage II and above
**Setting & Location**	Participants own home
**Study Perspective**	Payer and healthcare system perspective
**Comparators**	Pressure Injury Nutritional Care Bundle vs Standard Care
**Time Horizon**	1 year
**Choice of Health Outcomes**	QALY gained, reduction in wound area (percentage) and proportion of individuals with >40% reduction in wound area, prevention of new wounds, wound infections, mortality, hospitalization.
**Measurement of Effectiveness**	Based on a cluster RCT involving the largest population recruited for pressure injury
**Estimating Resources & Costs**	Cost data and resources collected alongside the RCT, with micro-costing done prior to start of RCT and CEA<br>Ministry of Health (Singapore) Published Benchmark Fees
**Currency, Price Date & Conversions**	Singapore Dollars (S$), 2023
**Analytical Methods**	Probabilistic sensitivity analysis<br>Scenario analysis

C. Consent Taking

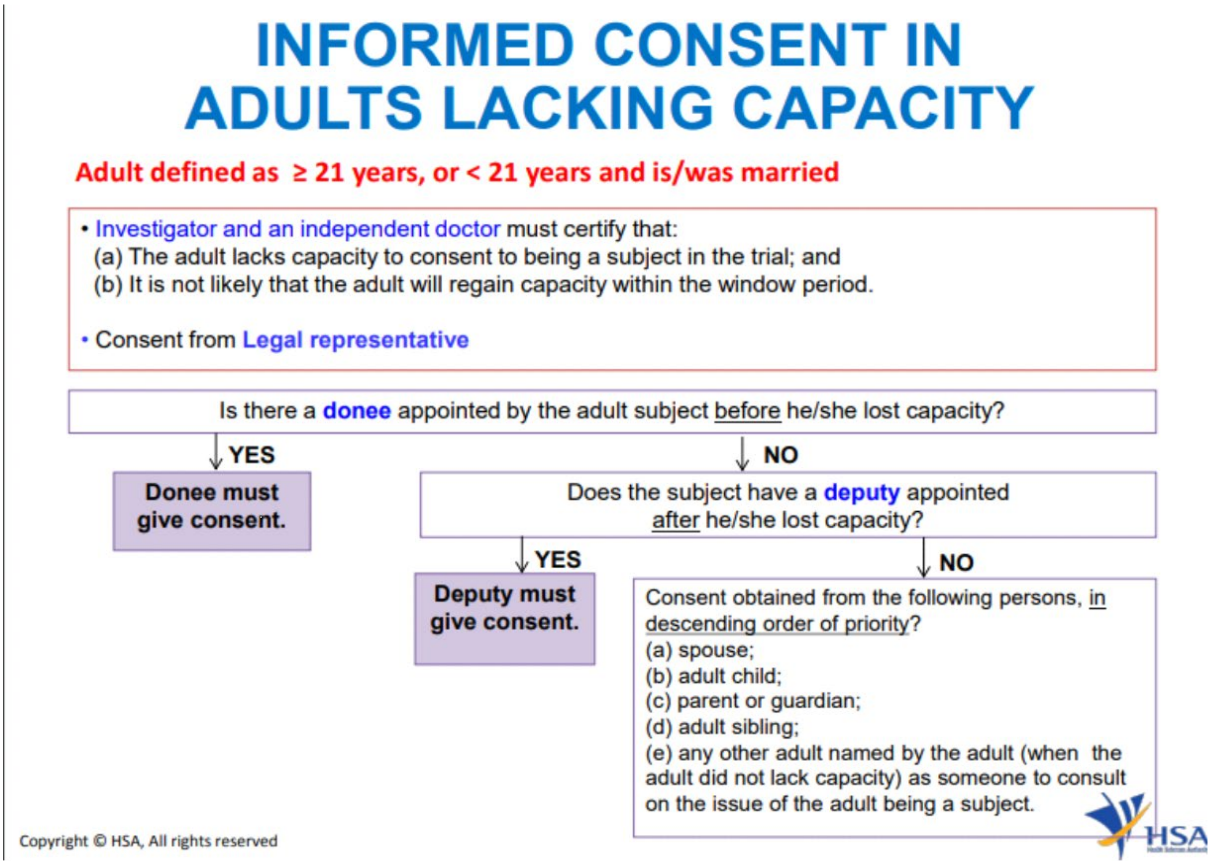

Figure S1. Informed Consent and Determining Legal Representative in Adults Lacking Capacity

D. Sample Participant Information Sheet and Consent Form

SingHealth

PARTICIPANT INFORMATION SHEET AND CONSENT FORM

You are being invited to participate in a research study. Your participation in this study is entirely voluntary. Before you take part in this research study, the study must be explained to you and you must be given the chance to ask questions. Your questions will be answered clearly and to your satisfaction. Please read carefully the information provided here. If you agree to participate, please sign the consent form. You will be given a copy of this document.

STUDY INFORMATION

Protocol Title:

Individualised Nutritional Care bundle for home nursing patients with pressure injuries – A Cluster-Randomised, Pragmatic Clinical Trial and Economic Evaluation. (INCA Trial)

Principal Investigator:

PURPOSE OF THE RESEARCH STUDY

The purpose of this study is to determine if an individualised nutritional care bundle is effective in pressure injury healing. We hope to learn if by providing a specialised support, pressure injury/injuries will heal at a faster rate or heal completely.

You were selected as a possible participant in this study because you have an existing stage II to unstageable pressure injury or pressure wound or bedsore.

This study targets to recruit 380 participants from Home Nursing Foundation.

STUDY PROCEDURES & YOUR RESPONSIBILITIES IN THIS STUDY

If you agree to take part in this study, you will be asked to follow a personalised nutritional therapy for wound healing, which includes nutritional education, and drinking of a specialised nutritional supplement containing L-Arginine (a type of protein building block). We will also measure your wound size to determine if it is improving over the study period. A photograph of the wound will be taken during each visit.

The following details the study procedure at each visit.

Study Visit 1 (Baseline):

You will be assessed to determine if you are suitable to participate in the study, if you do consent to participate, data will be collected with regards to your demographics and general health etc.

In addition to this, as part of the Research study,

- You will be provided with nutrition care bundle (Additional nutritional education provided by the community nurse) will be provided alongside your usual wound care.
- A photograph of your wound will also be taken to monitor the progress of the wound.
- If the Home Nursing Foundation nurse suspect that there may be a potential wound infection, 7mls (approximately 1½ teaspoon) of bloods for infectious markers (kidney and liver function test and C Reactive protein) may be obtained to rule out infection.
- You will be ask to keep a record of the 3 day food diary if you are on oral diet. and submit to the Home Nursing Foundation nurse during his/her next visit.
- You will be required to complete questionnaires, to assess for your health status. This should not take more than 15 minutes.
- You will be seen by a dietitian. This can be done via face to face home visit and/or teleconsultation.
- If you are on enteral feeding (ie feeding via nasal tube), has not been seen by any dietitian during your last hospital admission and required any adjustment in your feeding regime, the extra feeds will be provided during the 12 weeks of the intervention.
- You will be provided with a specialised nutritional supplement that contain L-Arginine, Vitamin C and Vitamin E which you will be required to take twice a day either through the oral route or through tube feeding (mixed in 100ml water) for 12 weeks.

<u>Study Visit 2 (D30):</u>

- Information regarding your current health status and any recent admission to the hospital will be collected.
- You will be seen by a dietitian. This can be done via face to face home visit and/or teleconsultation.
- A photograph of your wound will also be taken to monitor the progress of the wound.
- If the Home Nursing Foundation nurse suspect that there may be a potential wound infection, 7mls (approximately 1½ teaspoon) of bloods for infectious markers (kidney and liver function test and C Reactive protein) may be obtained to rule out infection.

<u>Study Visit 3 (D60):</u>

- Information regarding your current health status and any recent admission to the hospital will be collected.
- You will be seen by a dietitian. This can be done via face to face home visit and/or teleconsultation.
- A photograph of your wound will also be taken to monitor the progress of the wound.
- If the Home Nursing Foundation nurse suspect that there may be a potential wound infection, 7mls (approximately 1½ teaspoon) of bloods for infectious markers (kidney and liver function test and C Reactive protein) may be obtained to rule out infection.
- You will be ask to keep a record of the 3 day food diary if you are on oral diet. and submit to the Home Nursing Foundation nurse during his/her next visit.

<u>Study Visit 4 (D90):</u>

- Information regarding your current health status and any recent admission to the hospital will be collected.
- A photograph of your wound will also be taken to monitor the progress of the wound.
- If the Home Nursing Foundation nurse suspect that there may be a potential wound infection, 7mls (approximately 1½ teaspoon) of bloods for infectious markers (kidney and liver function test and C Reactive protein) may be obtained to rule out infection.

- You will be required to complete questionnaires, to assess for your health status. This should not take more than 15 minutes

Study Visit 5 (D180):

- Information regarding your current health status and any recent admission to the hospital will be collected.
- You will be required to complete questionnaires, to assess for your health status. This should not take more than 15 minutes

Study Visit 6 (Year 1):

- Information regarding your current health status and any recent admission to the hospital will be collected.
- A photograph of your wound will also be taken to monitor the progress of the wound.
- You will be required to complete questionnaires, to assess for your health status. This should not take more than 15 minutes

Your participation in the study will last for ONE YEAR. You will follow the nutritional advice provided by the dietitian and consume the L-Arginine/ Vitamin C/ Vitamin E supplement and any extra oral/ tube feeding supplements for about THREE MONTHS and be followed up for ONE YEAR.

Quality of Life Questionnaires (EQ5D) will be given to you to fill up. These will assess various aspects of quality of life.

The biological material will be tested for potential sources of infection and the samples will be tested in Singapore. It will not be used in research involving human-animal combinations, which is restricted by laws imposed by the Ministry of Health, Singapore.

The biological material will be tested for potential sources of infection and the samples will be tested in Singapore. It will not be used in research involving human-animal combinations, which is restricted by laws imposed by the Ministry of Health, Singapore.

If you agree to participate in this study, you should follow the advice and directions given to you by the study team.

WHAT IS NOT STANDARD CARE OR IS EXPERIMENTAL IN THIS STUDY

The study is being conducted because the individualised nutritional care bundle (consisting of nutritional education and supplementation with oral/tube feeding nutritional supplement and L-Arginine/ Vitamin C/ Vitamin E supplement, with usual nursing care for wound) is not yet proven to be a standard treatment in patients with pressure injury/ wound/ bedsore. We hope that your participation will help us to determine whether the individualised nutritional care bundle is equal or superior to existing treatment of standard wound nursing care.

Although oral/ tube feeding nutritional supplement and L-Arginine/ Vitamin C/ Vitamin E supplement may be part of standard medical care, in this study this / these procedure(s) are being performed for the purposes of the research, and are not part of your routine care

POSSIBLE RISKS, DISCOMFORTS OR INCONVENIENCES

Personal privacy and confidentiality:
This study uses health information that may affect your privacy. To protect your confidentiality, only a unique code number will be used to identify data and wound photographs that we collected from you.

As there will be a link between the code and your identifiable information, there is still a possibility of data breach. A data breach is when someone sees or uses data without permission. If there is a data breach, someone could see or use the data we have about you. Even without your name, there is a chance someone could figure out who you are. They could misuse your data. We believe the chance of this is very small, but it is not zero.

Questionnaires/ surveys/ interviews:
Some of the questions might make you feel uncomfortable or upset. You may refuse to answer any of the questions and/or take a break at any time during the study.

Collection of blood when there is a suspected wound infection:
Taking blood may cause momentary discomfort, pain, bleeding, bruising or swelling at the site of the needle stick. Rarely, taking blood may cause fainting or infection.

Drinking of supplementations:
Drinking of the L-Arginine/ Vitamin C/ Vitamin E supplement may cause gastrointestinal discomfort for some individuals, which include bloatedness, nausea, vomiting and diarrhea. Rarely, drinking of the L-Arginine/ Vitamin C/ Vitamin E supplement may cause allergic reactions

POTENTIAL BENEFITS

If you participate in this study, you may reasonably expect to benefit from the study intervention in the following way: faster wound healing rate and complete wound healing. Your participation may also add to the medical knowledge about the use of this intervention

IMPORTANT INFORMATION FOR FEMALE PARTICIPANTS

The effect of oral / tube feeding nutritional supplementation and L-Arginine/ Vitamin C/ Vitamin E supplement on a baby's development is not known. Therefore, pregnant and breast-feeding women may not take part in this study. Women who have a chance of becoming pregnant must have a negative pregnancy test at study entry and use birth control during the study. If you become pregnant during this study, you must stop taking the L-Arginine/ Vitamin C/ Vitamin E and call your doctor or the Principal Investigator immediately.

ALTERNATIVE PROCEDURES/ TREATMENTS IF YOU DO NOT PARTICIPATE IN THE STUDY

There is no alternative procedure or treatment to the study procedures. You can choose not to take part in this study. The study procedures will not be carried out.

COSTS & PAYMENTS IF PARTICIPATING IN THIS STUDY

There is no cost to you for participating in this research study.

If you take part in this study, the following will be performed at no charge to you:

- Nutritional education by community nurses and pamphlets,
- Blood tests if you have a suspected wound infection.
- Photograph of the wound
- Nutritional Supplement
- Additional nutritional feeds prescribe by the dietician if you are on enteral feeding (ie feeding via nasal tube), has not been seen by any dietitian during your last hospital admission and required any adjustment in your feeding regime, the extra feeds will be provided during the 12 weeks of the intervention.

These costs will be borne by Changi General Hospital/ Home Nursing Foundation/ Temasek Foundation.

The cost of your usual medical care (procedures, medications and doctor visits) will continue to be billed to you.

You will not receive any payments or reimbursements for taking part in this study.

INCIDENTAL FINDINGS

There will not be any incidental findings arising in this research. "Incidental findings" are findings that have potential health or reproductive importance to research participants like you and are discovered in the course of conducting the study, but are unrelated to the purposes, objectives or variables of the study.

WHAT HAPPENS TO THE SAMPLES COLLECTED FOR THE RESEARCH

The biological materials collected for this research study will be deemed to be donated to Changi General Hospital / Home Nursing Foundation as a gift. By agreeing to this, you give up your rights to the biological materials. If the use of your biological materials and/or your data results in intellectual property rights and commercial benefits, you will not receive any financial benefits or proprietary interest.

The biological materials will be used only for the purpose of this research and will be discarded or destroyed upon completion of the research study.

PARTICIPANT'S RIGHTS

Your participation in this study is entirely voluntary. You have a right to ask questions, which the study team will do their best to answer clearly and to your satisfaction.

In the event of any new information becoming available that may be relevant to your willingness to continue in this study, you (or your legal representative, if relevant) will be informed in a timely manner by the Principal Investigator or his/her representative and will be contacted for further consent if required.

WITHDRAWAL FROM STUDY

You are free to withdraw your consent and discontinue your participation in the study at any time, without your medical care being affected. If you decide to stop taking part in this study, you should tell the Principal Investigator.

If you withdraw from the study, or the study intervention is stopped for any reason,

- There will not be any anticipated consequences
- Contact the Clinical Research Coordinators or the Principal Investigators at the contact numbers listed below
- You will need to return all study-related supplies, including unused oral/ tube feeding nutritional supplements and L-Arginine/ Vitamin C/ Vitamin E supplement

However, any of your data that has been collected until the time of your withdrawal will be kept and analysed. The reason is to enable a complete and comprehensive evaluation of the study.

The biological materials that have been collected for the study will not be returned to you. However, you retain your right to ask the Principal Investigator to discard or destroy any remaining samples if they have not been anonymised and/or have not been used.

Your study doctor, the Principal Investigator of this study may stop your participation in the study at any time for one or more of the following reasons:

- Failure to follow the instructions of the Principal Investigator and/or study staff.
- The Principal Investigator decides that continuing your participation could be harmful to your health or safety.
- Pregnancy
- You require treatment not allowed in the study.
  The study is cancelled.

RESEARCH RELATED INJURY AND COMPENSATION

If you follow the directions of the Principal Investigator of this research study and you are injured due to the oral/ feeding tube nutritional supplements and L-Arginine/ Vitamin C/ Vitamin E supplement given under the plan for the research study, our institution will provide you with the appropriate medical treatment.

Payment for management of the normally expected consequences of your treatment (i.e. consequences of your treatment which are not caused by your participation in the research study) will not be provided.

You still have all your legal rights. Nothing said here about treatment or compensation in any way alters your right to recover damages where you can prove negligence.

CONFIDENTIALITY OF STUDY AND MEDICAL RECORDS

Your participation in this study will involve the collection of Personal Data. “Personal Data” means data about you which makes you identifiable (i) from such data or (ii) from that data and other information which an organisation has or likely to have access. Examples of personal data include name, national registration identity card (NRIC), nationality, passport information, date of birth, and telephone number.

Personal Data collected for this study will be kept confidential. Your study records and medical records, to the extent required by the applicable laws and regulations, will not be made publicly available. Only the study team will have access to the personal data being collected from you. In the event of any publication regarding this study, your identity will remain confidential.

However, the monitor(s), the auditor(s), the Institutional Review Board, and the regulatory authority(ies) will be granted direct access to your study records to verify study procedures and data, without making any of your information public.

By signing the Consent Form, you consent to (i) the collection, access to, use and storage of your Personal Data by Changi General Hospital/ Home Nursing Foundation, and (ii) the disclosure of such Personal Data to our authorised service providers and relevant third parties as mentioned above.

Any information containing your Personal Data that is collected for the purposes of this research will be stored in Singapore. To protect your identity, your Personal Data will be labelled with a unique code number. The code will be used in place of your name and other information that directly and easily identifies you. The study team will keep a separate file that links your code number to your Personal Data. This will be kept in a safe place with restricted access.

All data collected in this study are the property of Changi General Hospital/ Home Nursing Foundation. The data will be used for the purpose of this research study only.

By participating in this research study, you are confirming that you have read, understood and consent to the SingHealth Data Protection Policy, the full version of which is available at www.singhealth.com.sg/pdpa.

WHO HAS REVIEWED THE STUDY

This study has been reviewed by the SingHealth Centralised Institutional Review Board for ethics approval.

If you have questions about your rights as a participant, you can call the SingHealth Centralised Institutional Review Board at **8126 3660** during office hours (8:30 am to 5:30pm).

WHO TO CONTACT IF YOU HAVE QUESTIONS REGARDING THE STUDY

If you have questions about this research study or in the case of any injuries during the course of this study, you may contact:

Principal Investigator

Department of Dietetics, Changi General Hospital
Mobile:
Office:

Nursing Department, Home Nursing Foundation
Mobile:
Office:

If you have any feedback about this research study, you may contact the Principal Investigator or the SingHealth Centralised Institutional Review Board.

CONSENT FORM FOR RESEARCH STUDY

Protocol Title:
Individualised Nutritional Care bundle for home nursing patients with pressure injuries – A Cluster-Randomised, Pragmatic Clinical Trial and Economic Evaluation. (INCA Trial)

Principal Investigator:
Mr ABC
Department of Dietetics, Changi General Hospital

Ms XYZ
Nursing Department, Home Nursing Foundation

I agree to participate in the research study as described and on the terms set out in the Participant Information Sheet.

The nature, risks and benefits of the study have been explained clearly to me and I fully understand them.

I understand the purpose and procedures of this study. I have been given the Participant Information Sheet and the opportunity to discuss and ask questions about this study and am satisfied with the information provided to me.

I understand that my participation is voluntary and that I am free to withdraw at any time, without giving any reasons and without my medical care being affected.

By participating in this research study, I confirm that I have read, understood and consent to the SingHealth Data Protection Policy.

To be completed by parent / legal guardian / legal representative, where applicable

I hereby give consent for __________________________ (Name of Participant) to participate in the research study. The nature, risks and benefits of the study have been explained clearly to me and I fully understand them.

I confirm that I have read, understood and consent to the SingHealth Data Protection Policy.

______________________	______________________________	_______________
Name of participant's parent/ legal guardian/ legal representative	Signature/Thumbprint (Right / Left)	Date of signing

To be completed by translator, if required

The study has been explained to the participant/ legal representative in

__________________________________ by _______________________________________.
Language — Name of translator

To be completed by witness, where applicable

I, the undersigned, certify that:

- I am 21 years of age or older.
- To the best of my knowledge, the participant or the participant's legal representative signing this informed consent form had the study fully explained to him/her in a language understood by him/ her and clearly understands the nature, risks and benefits of the participant's participation in the study.
- I have taken reasonable steps to ascertain the identity of the participant or the participant's legal representative giving the consent.
- I have taken reasonable steps to ascertain that the consent has been given voluntarily without any coercion or intimidation.

Witnessed by: ________________________________ ___________________

Name of witness | Date of signing

____

Signature of witness

1. An impartial witness (who is 21 years of age or older, has mental capacity, who is independent of the research study, and cannot be unfairly influenced by people involved with the research study) should be present during the entire informed consent discussion if a participant or the participant's legal representative is unable to read, and/or sign and date on the consent form (i.e. using the participant's or legal representative's thumbprint). After the written consent form and any written information to be provided to participant is read and explained to the participant or the participant's legal representative, and after the participant or the participant's legal representative has orally consented to the participant's participation in the study and, if capable of doing so, has signed and personally dated the consent form, the witness should sign and personally date the consent form. This is applicable for Clinical Trials regulated by HSA and Human Biomedical Research under the HBRA.

2. For HBRA studies, the witness may be a member of the team carrying out the research only if a participant or the participant's legal representative is able to read, sign and date on the consent form.

Investigator's Statement

I, the undersigned, certify to the best of my knowledge that the participant/ participant's legal representative signing this consent form had the study fully explained to him/her and clearly understands the nature, risks and benefits of the participant's participation in the study.

________________________ ________________________ ________________

Name of Investigator/ Person obtaining consent | Signature | Date

SPIRIT-Outcomes 2022 Checklist (for combined completion of SPIRIT 2013 and SPIRIT-Outcomes 2022 items)[a]

Section	Item No.	SPIRIT 2013 Item	SPIRIT-Outcomes 2022 item	Location Reported[b]
**Administrative information**				
Title	1	Descriptive title identifying the study design, population, interventions, and, if applicable, trial acronym	-	Page 1
Trial registration	2a	Trial identifier and registry name. If not yet registered, name of intended registry	NCT06078488, 10th October 2023	www.clinicaltrials.gov
	2b	All items from the World Health Organization Trial Registration Data Set	-	Page 1 - 15
Protocol version	3	Date and version identifier	-	Page 18
Funding	4	Sources and types of financial, material, and other support	-	Page 18
Roles and responsibilities	5a	Names, affiliations, and roles of protocol contributors	-	Page 19
	5b	Name and contact information for the trial sponsor	-	Page 18
	5c	Role of study sponsor and funders, if any, in study design; collection, management, analysis, and interpretation of data; writing of the report; and the decision to submit the report for publication, including whether they will have ultimate authority over any of these activities	-	Page 18
	5d	Composition, roles, and responsibilities of the coordinating centre, steering committee, endpoint adjudication committee, data management team, and other individuals or groups overseeing the trial, if applicable (see Item 21a for data monitoring committee)	-	Page 18
**Introduction**				
Background and rationale	6a	Description of research question and justification for undertaking the trial, including summary of relevant studies (published and unpublished) examining benefits and harms for each intervention	-	Page 3-9
	6b	Explanation for choice of comparators	-	Page 9
Objectives	7	Specific objectives or hypotheses	-	Page 3

Section	Item No.	SPIRIT 2013 Item	SPIRIT-Outcomes 2022 item	Location Reported[b]
Trial design	8	Description of trial design including type of trial (eg, parallel group, crossover, factorial, single group), allocation ratio, and framework (eg, superiority, equivalence, noninferiority, exploratory)	-	Page 3
**Methods: Participants, interventions, and outcomes**				
Study setting	9	Description of study settings (eg, community clinic, academic hospital) and list of countries where data will be collected. Reference to where list of study sites can be obtained	-	Page 3 - 4
Eligibility criteria	10	Inclusion and exclusion criteria for participants. If applicable, eligibility criteria for study centres and individuals who will perform the interventions (eg, surgeons, psychotherapists)	-	Page 3 and Table 1
Interventions	11a	Interventions for each group with sufficient detail to allow replication, including how and when they will be administered (for specific guidance see TIDieR checklist and guide)	-	Page 5
	11b	Criteria for discontinuing or modifying allocated interventions for a given trial participant (eg, drug dose change in response to harms, participant request, or improving/worsening disease)	-	Page 8 - 9
	11c	Strategies to improve adherence to intervention protocols, and any procedures for monitoring adherence (eg, drug tablet return, laboratory tests)	-	Page 6
	11d	Relevant concomitant care and interventions that are permitted or prohibited during the trial	-	Page 6
Outcomes	12	Primary, secondary, and other outcomes, including the specific measurement variable (eg, systolic blood pressure), analysis metric (eg, change from baseline, final value, time to event), method of aggregation (eg, median, proportion), and time point for each outcome. Explanation of the clinical relevance of chosen efficacy and harm outcomes is strongly recommended	-	Page 9-11

Section	Item No.	SPIRIT 2013 Item	SPIRIT-Outcomes 2022 item	Location Reported[b]
	12.1		Provide a rationale for the selection of the domain for the trial's primary outcome	Page 7
	12.2		If the analysis metric for the primary outcome represents within-participant change, define and justify the minimal important change in individuals	Page 7
	12.3		If the outcome data collected are continuous but will be analyzed as categorical (method of aggregation), specify the cutoff values to be used	NA
	12.4		If outcome assessments will be performed at several time points after randomization, state the time points that will be used for analysis	Page 7
	12.5		If a composite outcome is used, define all individual components of the composite outcome	NA
Participant timeline	13	Time schedule of enrolment, interventions (including any run-ins and washouts), assessments, and visits for participants. A schematic diagram is highly recommended (see Figure)	-	Table 2 and 3
Sample size	14	Estimated number of participants needed to achieve study objectives and how it was determined, including clinical and statistical assumptions supporting any sample size calculations	-	Page 3 – 4<br>Supplementary material Figure S1
	14.1		Define and justify the target difference between treatment groups (eg, the minimal important difference)	Page 4-5
Recruitment	15	Strategies for achieving adequate participant enrolment to reach target sample size	-	Page 6

Section	Item No.	SPIRIT 2013 Item	SPIRIT-Outcomes 2022 item	Location Reported[b]
**Methods: Assignment of interventions (for controlled trials)**				
Allocation:				
Sequence generation	16a	Method of generating the allocation sequence (eg, computer-generated random numbers), and list of any factors for stratification. To reduce predictability of a random sequence, details of any planned restriction (eg, blocking) should be provided in a separate document that is unavailable to those who enrol participants or assign interventions	-	Page 6-7

Section	Item No.	SPIRIT 2013 Item	SPIRIT-Outcomes 2022 item	Location Reported[b]
Allocation concealment mechanism	16b	Mechanism of implementing the allocation sequence (eg, central telephone; sequentially numbered, opaque, sealed envelopes), describing any steps to conceal the sequence until interventions are assigned	-	Page 7
Implementation	16c	Who will generate the allocation sequence, who will enrol participants, and who will assign participants to interventions	-	Page 7
Blinding (masking)	17a	Who will be blinded after assignment to interventions (eg, trial participants, care providers, outcome assessors, data analysts), and how	-	Page 7
	17b	If blinded, circumstances under which unblinding is permissible, and procedure for revealing a participant's allocated intervention during the trial	-	Page 15
**Methods: Data collection, management, and analysis**				
Data collection methods	18a	Plans for assessment and collection of outcome, baseline, and other trial data, including any related processes to promote data quality (eg, duplicate measurements, training of assessors) and a description of study instruments (eg, questionnaires, laboratory tests) along with their reliability and validity, if known. Reference to where data collection forms can be found, if not in the protocol	-	Page 10 - 14

	18a.1		Describe what is known about the responsiveness of the study instruments in a population similar to the study sample	Page 4
	18a.2		Describe who will assess the outcome (eg, nurse, parent)	Page 14
	18b	Plans to promote participant retention and complete follow-up, including list of any outcome data to be collected for participants who discontinue or deviate from intervention protocols	-	Page 10

Section	Item No.	SPIRIT 2013 Item	SPIRIT-Outcomes 2022 item	Location Reported[b]
Data management	19	Plans for data entry, coding, security, and storage, including any related processes to promote data quality (eg, double data entry; range checks for data values). Reference to where details of data management procedures can be found, if not in the protocol	-	Page 14
Statistical methods	20a	Statistical methods for analysing primary and secondary outcomes. Reference to where other details of the statistical analysis plan can be found, if not in the protocol	-	Page 19-20
	20a.1		Describe any planned methods to account for multiplicity in the analysis or interpretation of the primary and secondary outcomes (eg, coprimary outcomes, same outcome assessed at multiple time points, or subgroup analyses of an outcome)	Page 20
	20b	Methods for any additional analyses (eg, subgroup and adjusted analyses)	-	Page 19-20
	20c	Definition of analysis population relating to protocol non-adherence (eg, as randomised analysis), and any statistical methods to handle missing data (eg, multiple imputation)	-	Page 19-20

Methods: Monitoring				
Data monitoring	21a	Composition of data monitoring committee (DMC); summary of its role and reporting structure; statement of whether it is independent from the sponsor and competing interests; and reference to where further details about its charter can be found, if not in the protocol. Alternatively, an explanation of why a DMC is not needed	-	Page 23-24
	21b	Description of any interim analyses and stopping guidelines, including who will have access to these interim results and make the final decision to terminate the trial	-	Page 23-24
Harms	22	Plans for collecting, assessing, reporting, and managing solicited and spontaneously reported adverse events and other unintended effects of trial interventions or trial conduct	-	Page 23-24

Section	Item No.	SPIRIT 2013 Item	SPIRIT-Outcomes 2022 item	Location Reported[b]
Auditing	23	Frequency and procedures for auditing trial conduct, if any, and whether the process will be independent from investigators and the sponsor	-	Page 23-24
**Ethics and dissemination**				
Research ethics approval	24	Plans for seeking research ethics committee/institutional review board (REC/IRB) approval	-	Page 26
Protocol amendments	25	Plans for communicating important protocol modifications (eg, changes to eligibility criteria, outcomes, analyses) to relevant parties (eg, investigators, REC/IRBs, trial participants, trial registries, journals, regulators)	-	Page 26
Consent or assent	26a	Who will obtain informed consent or assent from potential trial participants or authorised surrogates, and how (see Item 32)	-	Supplementary Material Figure S2
	26b	Additional consent provisions for collection and use of participant data and biological specimens in ancillary studies, if applicable	-	NA
Confidentiality	27	How personal information about potential and enrolled participants will be collected, shared, and maintained in order to protect confidentiality before, during, and after the trial	-	Page 26
Declaration of interests	28	Financial and other competing interests for principal investigators for the overall trial and each study site	-	Page 26
Access to data	29	Statement of who will have access to the final trial dataset, and disclosure of contractual agreements that limit such access for investigators	-	Page 26
Ancillary and post-trial care	30	Provisions, if any, for ancillary and post-trial care, and for compensation to those who suffer harm from trial participation	-	Page 24
Dissemination policy	31a	Plans for investigators and sponsor to communicate trial results to participants, healthcare professionals, the public, and other relevant groups (eg, via publication, reporting in results databases, or other data sharing arrangements), including any publication restrictions	-	Page 26
	31b	Authorship eligibility guidelines and any intended use of professional writers	-	Page 27

Section	Item No.	SPIRIT 2013 Item	SPIRIT-Outcomes 2022 item	Location Reported[b]
	31c	Plans, if any, for granting public access to the full protocol, participant-level dataset, and statistical code	-	Page 26
**Appendices**				
Informed consent materials	32	Model consent form and other related documentation given to participants and authorised surrogates	-	Supplementary Material
Biological specimens	33	Plans for collection, laboratory evaluation, and storage of biological specimens for genetic or molecular analysis in the current trial and for future use in ancillary studies, if applicable	-	NA

[a]It is strongly recommended that this checklist be read in conjunction with the SPIRIT (Standard Protocol Items: Recommendations for Interventional Trials) Statement paper for important clarification on the items. Amendments to the protocol should be tracked and dated. The SPIRIT checklist is copyrighted by the SPIRIT Group under the Creative Commons "Attribution-NonCommercial-NoDerivs 3.0 Unported" license and is reproduced with permission.
[b]Indicates page numbers and/or manuscript location: to be completed by authors.

[42]

TIDieR Template for Intervention Description and Replication

The TIDieR (Template for Intervention Description and Replication) Checklist*:

Information to include when describing an intervention and the location of the information

Item number	Item	Where located ** Primary paper (page or appendix number)	Other † (details)
	**BRIEF NAME**		
**1.**	Provide the name or a phrase that describes the intervention.	1	
	**WHY**		
**2.**	Describe any rationale, theory, or goal of the elements essential to the intervention.	3	
	**WHAT**		
**3.**	Materials: Describe any physical or informational materials used in the intervention, including those provided to participants or used in intervention delivery or in training of intervention providers. Provide information on where the materials can be accessed (e.g. online appendix, URL).	8	Supplementary Material
**4.**	Procedures: Describe each of the procedures, activities, and/or processes used in the intervention, including any enabling or support activities.	8-9	
	**WHO PROVIDED**		
**5.**	For each category of intervention provider (e.g. psychologist, nursing assistant), describe their expertise, background and any specific training given.	8	
	**HOW**		

**6.**	Describe the modes of delivery (e.g. face-to-face or by some other mechanism, such as internet or telephone) of the intervention and whether it was provided individually or in a group.	8-9	
	**WHERE**		
**7.**	Describe the type(s) of location(s) where the intervention occurred, including any necessary infrastructure or relevant features.	8-9	
	**WHEN and HOW MUCH**		
**8.**	Describe the number of times the intervention was delivered and over what period of time including the number of sessions, their schedule, and their duration, intensity or dose.		Tables 2 & 3
	**TAILORING**		
**9.**	If the intervention was planned to be personalised, titrated or adapted, then describe what, why, when, and how.	9	
	**MODIFICATIONS**		
**10.**ǂ	If the intervention was modified during the course of the study, describe the changes (what, why, when, and how).	NA	
	**HOW WELL**		
**11.**	Planned: If intervention adherence or fidelity was assessed, describe how and by whom, and if any strategies were used to maintain or improve fidelity, describe them.	8	
**12.**ǂ	Actual: If intervention adherence or fidelity was assessed, describe the extent to which the intervention was delivered as planned.	NA	

** **Authors -** use N/A if an item is not applicable for the intervention being described. **Reviewers** – use '?' if information about the element is not reported/not sufficiently reported.

† If the information is not provided in the primary paper, give details of where this information is available. This may include locations such as a published protocol or other published papers (provide citation details) or a website (provide the URL).
ǂ If completing the TIDieR checklist for a protocol, these items are not relevant to the protocol and cannot be described until the study is complete.

* We strongly recommend using this checklist in conjunction with the TIDieR guide (see *BMJ* 2014;348:g1687) which contains an explanation and elaboration for each item.
* The focus of TIDieR is on reporting details of the intervention elements (and where relevant, comparison elements) of a study. Other elements and methodological features of studies are covered by other reporting statements and checklists and have not been duplicated as part of the TIDieR checklist. When a **randomised trial** is being reported, the TIDieR checklist should be used in conjunction with the CONSORT statement (see www.consort-statement.org) as an extension of **Item 5 of the CONSORT 2010 Statement.** When a **clinical trial protocol** is being reported, the TIDieR checklist should be used in conjunction with the SPIRIT statement as an extension of **Item 11 of the SPIRIT 2013 Statement** (see www.spirit-statement.org). For alternate study designs, TIDieR can be used in conjunction with the appropriate checklist for that study design (see www.equator-network.org
